# Atrial Myxoma Presenting as Myocardial Infarction Diagnosed by Echocardiography, Managed Endoscopically with Robot-Assisted Surgery

**DOI:** 10.7759/cureus.484

**Published:** 2016-02-07

**Authors:** Aadel A Chaudhuri, Charles, Jr. Simmons, Douglas Ellison, James Hemp, Kiyon Chung

**Affiliations:** 1 Department of Radiation Oncology, Stanford University School of Medicine; 2 Emergency Medicine, Scripps Mercy Hospital; 3 Pathology, Scripps Mercy Hospital; 4 Cardiothoracic Surgery, Scripps Mercy Hospital; 5 Cardiology, Scripps Mercy Hospital

**Keywords:** atrial myxoma, myocardial infarction, echocardiography, non-st elevation myocardial infarction, nstemi, robotic surgery, endoscopic surgery

## Abstract

Atrial myxomatous embolization into the coronary arteries is a rare event. Management of large myxomas is usually via surgical resection involving a median sternotomy. Echocardiography is not a routine part of non-ST-elevation myocardial infarction (NSTEMI) management. Here, we present the case of a 70-year-old Caucasian man with a history of hypertension and hyperlipidemia who presented to the emergency department with an NSTEMI. Transthoracic echocardiogram and transesophageal echocardiogram revealed a large and highly mobile atrial mass, traversing through the mitral valve orifice during diastole. Coronary angiography revealed a focal 60% lesion in the right coronary artery and no other significant obstructive coronary artery disease, suggesting that the cause of his presentation was tumor embolization into the coronary circulation. The patient underwent robot-assisted endoscopic resection of his atrial mass and was discharged in stable condition on postoperative day 2. Pathology revealed atrial myxoma. To our knowledge, this is the first reported case of an atrial myxoma presenting with an NSTEMI and managed with a robot-assisted endoscopic approach. This case also highlights the importance of routine early echocardiography in patients presenting with NSTEMI.

## Introduction

Atrial myxomas are cardiac tumors that often present with symptoms of congestive heart failure or systemic tumor embolization [1-2]. Myxomatous embolization into the coronary arteries, however, is a rare event [3-10]. Here, we present the case of a patient who presented with a large left atrial myxoma with evidence of embolization into the coronary arteries causing a non-ST-elevation myocardial infarction (NSTEMI). We show that in cases such as this one it is prudent to obtain a chest echocardiogram prior to coronary angiography for optimal medical and surgical management. We managed this patient with robot-assisted endoscopic surgery, showing this is a viable technique in patients with left atrial myxoma presenting with NSTEMI.

## Case presentation

A 70-year-old Caucasian male with a history of hypertension and hyperlipidemia presented to our emergency department complaining of new-onset arm tingling and chest tightness that began while he was exercising. He had no known history of coronary artery disease or angina. The patient’s vital signs on presentation were significant for an elevated blood pressure of 184/95 but were otherwise within normal limits. His physical exam, including a full cardiac and full neurologic exam, was unremarkable except for a Grade 2 mid-diastolic murmur. The remainder of a 14-point review of systems was negative. He had no known history of hyperlipidemia, diabetes, or smoking. EKG displayed a normal sinus rhythm. Cardiac enzymes were, however, elevated with a troponin at 3.18, CK-MB at 10.6, and CK at 279. Given the evidence of an acute NSTEMI, we administered aspirin, beta blockers, nitroglycerin, and a statin. Informed patient consent was obtained. He was placed on a heparin drip and admitted to the hospital.

**Figure 1:**
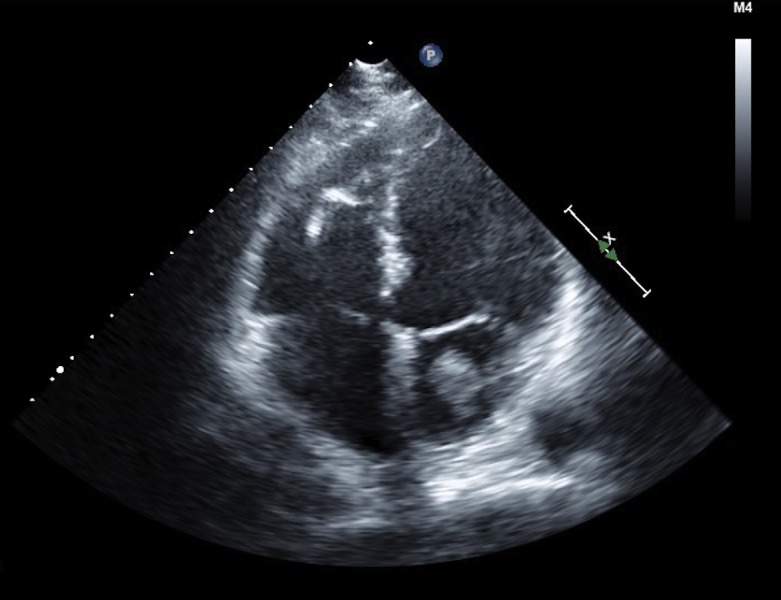
Transthoracic echocardiogram, 4-chamber view, demonstrates a left atrial mass measuring up to 5 cm in length.

The patient was scheduled for coronary angiography for the next day, and an echocardiogram was first performed. To our surprise, the trans-thoracic 2D chest echocardiogram showed a large, highly mobile left atrial mass extending through the mitral valve into the left ventricle during diastole (Figure 1, Video 1).

**Video 1:** Transthoracic echocardiogram, 4-chamber view, reveals a large and highly mobile left atrial mass that extends through the mitral valve into the left ventricle during diastole.

**Figure 2:**
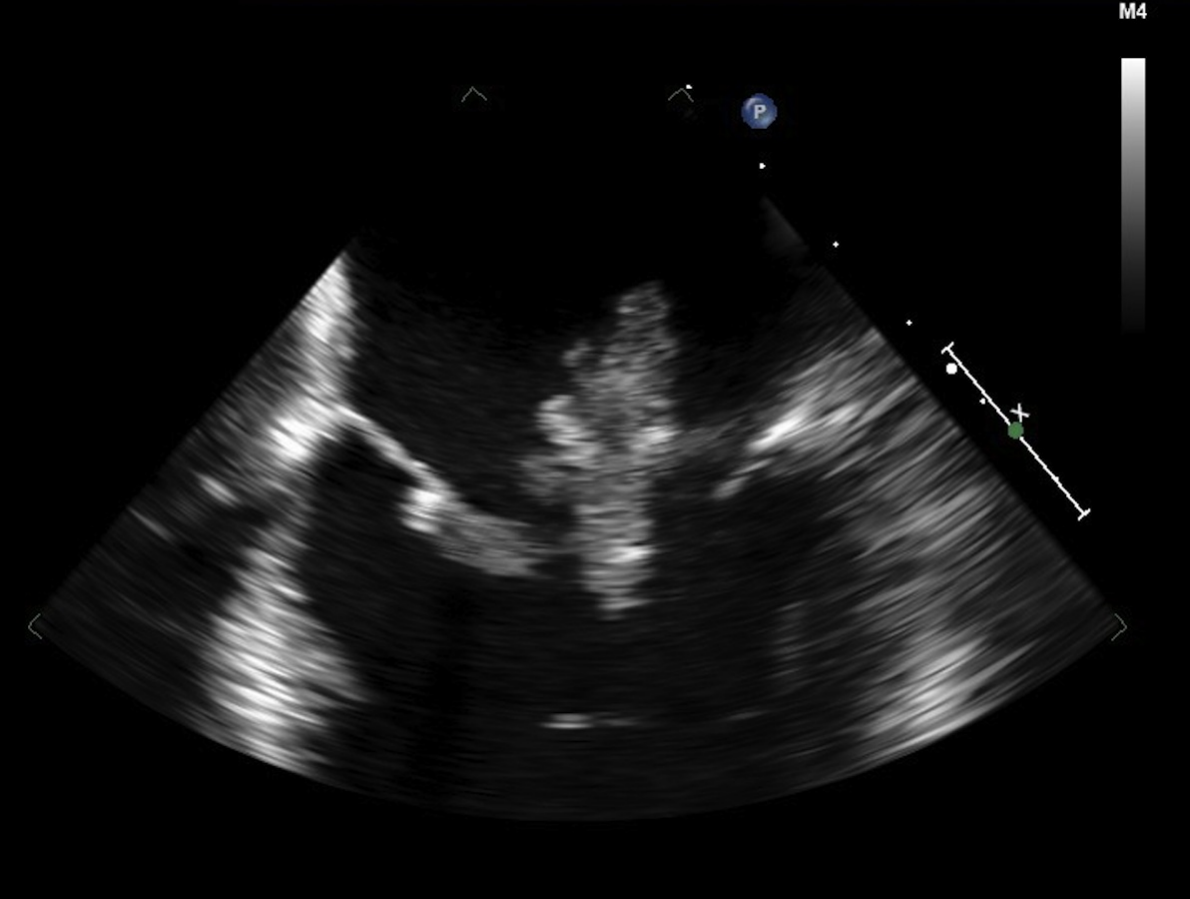
2D transesophageal echocardiogram shows a large left atrial mass that protrudes through the mitral valve during diastole.

**Figure 3:**
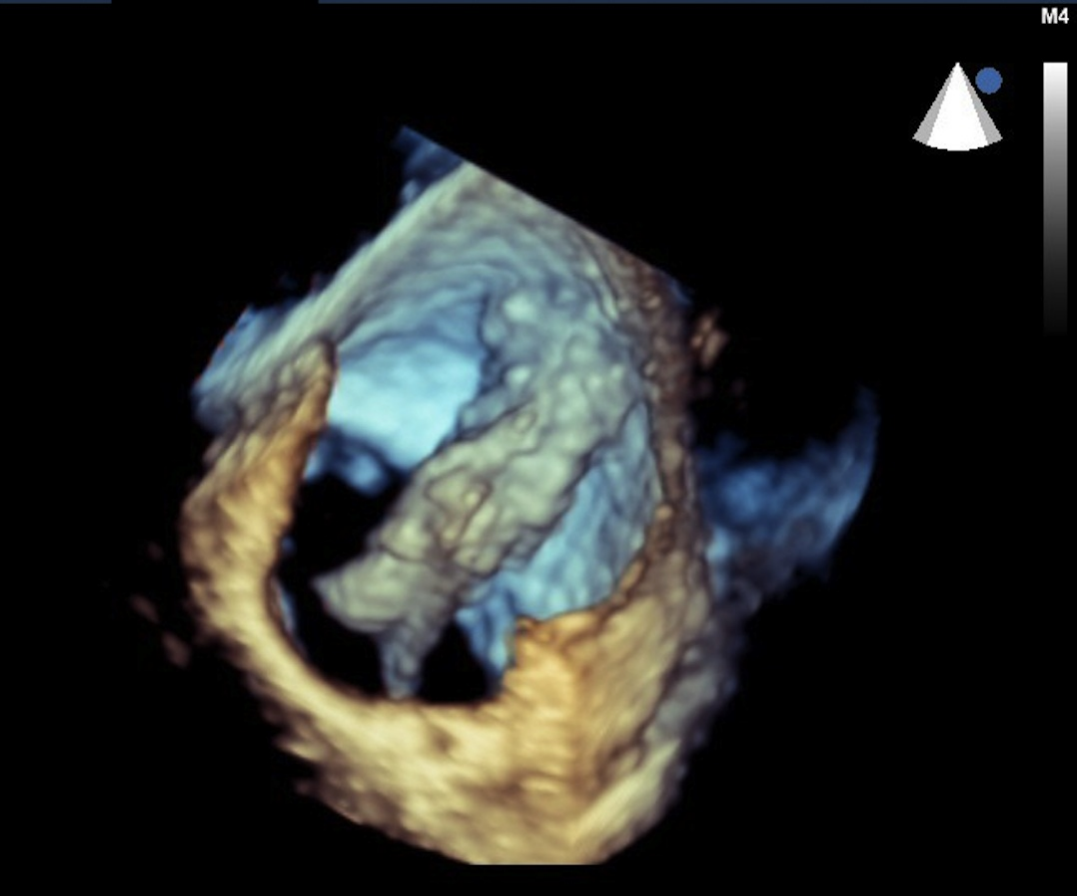
3D transesophageal echocardiogram shows a large left atrial mass and its attachment point on the posterior left atrial wall.

The echocardiogram was otherwise normal, with preserved left ventricular function, no wall motion abnormalities, and normal valvular function. To better assess his large left atrial mass, we next performed a transesophageal echocardiogram with 3D reconstruction (Figures 2-3, Videos 2-3).

**Video 2:** 2D transesophageal echocardiogram demonstrates a large and highly mobile left atrial mass that protrudes through the mitral valve during diastole.

**Video 3:** 3D transesophageal echocardiogram demonstrates a large and highly mobile left atrial mass, swinging from its attachment point on the posterior left atrial wall.

This confirmed the finding of a large, highly mobile left atrial mass measuring at least 4.7 cm in length. The mass arose from the posterior aspect of the left atrial septum, extended across the atrium, and traversed the mitral valve into the left ventricle during diastole. It appeared to be most consistent with an atrial myxoma.

**Figure 4:**
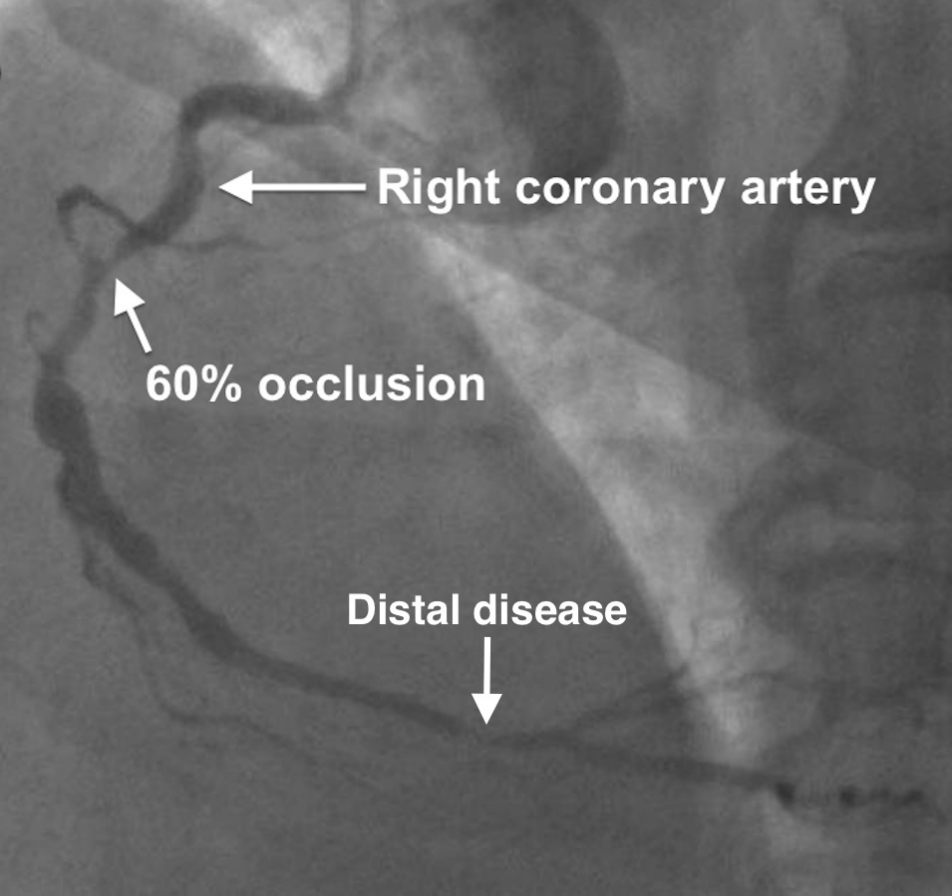
Coronary angiography reveals a focal 60% occlusion at the proximal middle portion of the right coronary artery (RCA) and some distal RCA disease.

Given this patient was presenting with a highly mobile, large left atrial mass in the setting of NSTEMI, we posited that a portion of this mass had embolized into his coronary arteries, causing a myocardial infarction. Coronary angiography showed single vessel disease in the proximal mid-portion of the right coronary artery, which contained 60% of a focal lesion, and some distal right coronary artery disease (Figure 4). Thus, the coronary angiography results supported our initial theory.

**Figure 5:**
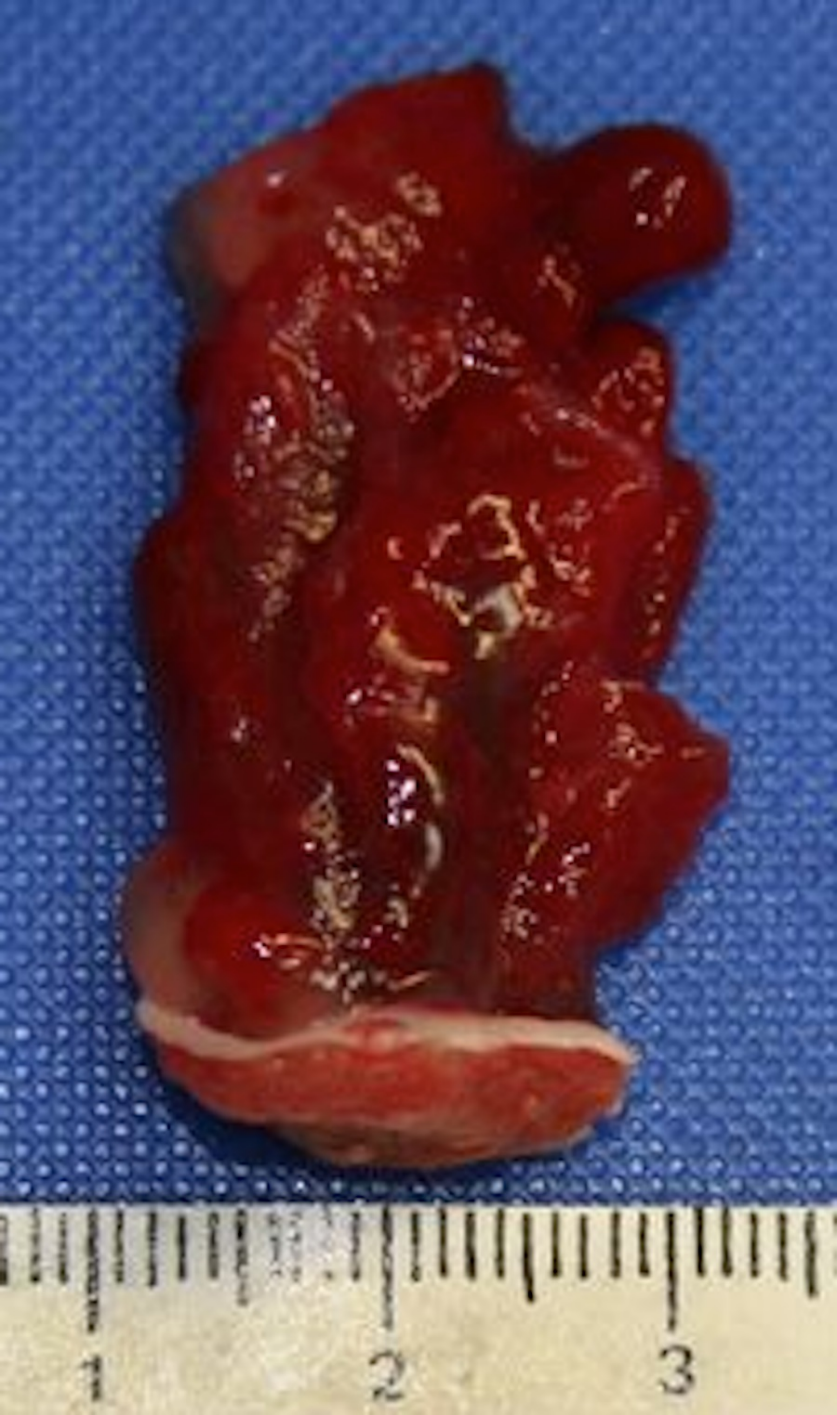
Gross pathology demonstrates a multilobulated atrial myxoma attached to the inner surface of the atrial myocardium.

The patient was seen by cardiothoracic surgery, who felt the patient would be a good candidate for robot-assisted endoscopic surgery. He then underwent robot-assisted endoscopic surgical resection of his left atrial myxoma using the da Vinci^®^ robot-assisted surgical system (Intuitive Surgical, Inc., Sunnyvale, CA). A working port, three robotic ports, and three suture retraction ports were created in the chest. The heart was arrested with cold blood cardioplegia and a generous left atriotomy was made robotically. The myxoma and its attachment point on the left atrial wall were easily seen. A full-thickness excision of the left atrial wall was accomplished and the myxoma excised. Gross pathology review of the mass demonstrated a 1.4 x 0.9 cm (base) x 3.5 cm (height) multilobulated, red-colored, semi-translucent polypoid mass protruding from the inner surface of the atrial myocardium (Figure 5).

**Figure 6:**
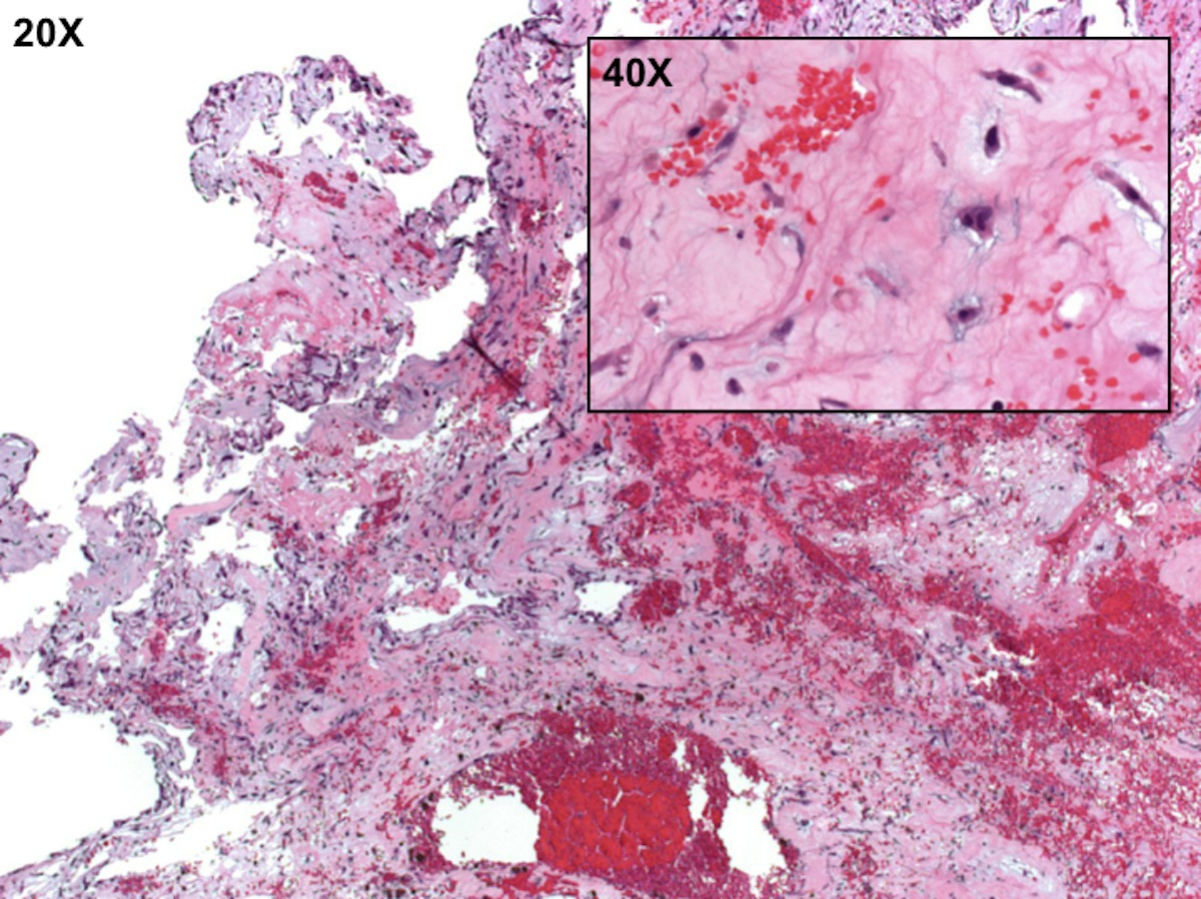
Microscopic pathology shows an atrial myxoma with mildly pleomorphic spindled and epithelioid cells in myxoid stroma with secondary hemorrhage, congestion, and adherent thrombus.

Microscopic pathology review demonstrated mildly pleomorphic spindled and epithelioid cells in a myxoid stroma with secondary hemorrhage, congestion, and adherent thrombus (Figure 6), findings consistent with an atrial myxoma.

The patient tolerated the surgical procedure well and was discharged on postoperative day 2 after an uncomplicated recovery. He continued to do well with no cardiopulmonary symptomatology at his clinic visits three and five weeks after hospital discharge. Moreover, a myocardial stress test performed one month postoperatively revealed no evidence of myocardial ischemia or infarction, supporting that his right coronary artery (RCA) stenosis was not hemodynamically significant.

## Discussion

We described here a case of a hemodynamically stable patient presenting with an NSTEMI and incidentally found to have a large, highly mobile left atrial myxoma with coronary angiographic evidence suggesting that the myocardial infarction resulted from myxomatous embolization into the coronary arteries. Vigorous cardiac contractions during exercise might have provided the impetus for a portion of the large and highly mobile atrial myxoma to embolize.

Echocardiography is not always performed prior to coronary angiography in the setting of myocardial infarction [11]. If this patient had not undergone echocardiography prior to coronary angiography, then his NSTEMI could have easily been attributed to the 60% focal RCA lesion. He would likely have been treated with a stent to the RCA and would require dual antiplatelet therapy for his coronary stent, making a bleeding risk in subsequent surgery much higher. In some cases, echocardiography may even be skipped if angiography is performed first and left ventriculography shows normal systolic function [11]. Given the high embolic potential of the myxoma in our case, failing to make the diagnosis during this hospitalization could have led to serious consequences. Our recommendation of the routine use of echocardiography for evaluation of a suspected myocardial infarction to diagnose a suspected cardiac mass is in accordance with the published appropriate use criteria for echocardiography [12].

While robot-assisted endoscopic surgery is becoming more utilized in cardiothoracic surgery [13], there are still only a limited number of reports showing its use for resection of atrial myxomas [14-19]. Notably, the patients in the literature who underwent this technique for atrial myxoma resection did not also present with myocardial infarction or arterial tumor embolization [14-19]. We confirm here that robot-assisted surgery is an effective method for endoscopic resection of atrial myxoma. Moreover, to our knowledge, this is the first reported case of a patient with both left atrial myxoma and NSTEMI who was successfully managed with robot-assisted endoscopic surgery. 

## Conclusions

We present a patient with an NSTEMI, found to have a large left atrial myxoma on echocardiography, which was resected endoscopically using a robot-assisted technique. Our case demonstrates the importance of routine early echocardiography in patients presenting with NSTEMI and indicates that robot-assisted endoscopic surgery can be used for left atrial myxoma resection in a hemodynamically stable NSTEMI patient.

## References

[1] Pinede L, Duhaut P, Loire R (2001). Clinical presentation of left atrial cardiac myxoma. A series of 112 consecutive cases. Medicine (Baltimore).

[2] Swartz MF, Lutz CJ, Chandan VS, Landas S, Fink GW (2006). Atrial myxomas: Pathologic types, tumor location, and presenting symptoms. J Card Surg.

[3] Konagai N, Cho M, Nakamura K, Shigematsu H (2010). Left atrial myxoma as a cause of acute myocardial infarction. Tex Heart Inst J.

[4] Butany J (2006). Left atrial myxoma presenting as acute inferior wall infarction--a case report. J Card Surg.

[5] Sankar NM, Vaidyanathan RK, Prasad GN, Cherian KM (2006). Left atrial myxoma presenting as acute inferior wall infarction-a case report. J Card Surg.

[6] Demir M, Akpinar O, Acarturk E (2005). Atrial myxoma: an unusual cause of myocardial infarction. Tex Heart Inst J.

[7] Ozaydin M, Dogan A, Altinbas A (2005). Left atrial myxoma presenting with acute myocardial infarction--a case report. Angiology.

[8] Tóth C, Lengyel M (2002). Images in cardiology: acute myocardial infarction as first manifestation of left atrial myxoma. Acta Cardiol.

[9] Panos A, Kalangos A, Sztajzel J (1997). Left atrial myxoma presenting with myocardial infarction. Case report and review of the literature. Int J Cardiol.

[10] Wenger NK, Bauer S (1958). Coronary embolism: review of the literature and presentation of fifteen cases. Am J Med.

[11] Levine GN, Bates ER, Blankenship JC, Bailey SR, Bittl JA, Cercek B, Chambers CE, Ellis SG, Guyton RA, Hollenberg SM, Khot UN, Lange RA, Mauri L, Mehran R, Moussa ID, Mukherjee D, Nallamothu BK, Ting HH; American College of Cardiology Foundation; American Heart Association Task Force on Practice Guidelines; Society for Cardiovascular Angiography and Interventions (2011). 2011 ACCF/AHA/SCAI Guideline for Percutaneous Coronary Intervention. A report of the American College of Cardiology Foundation/American Heart Association Task Force on Practice Guidelines and the Society for Cardiovascular Angiography and Interventions. J Am Coll Cardiol.

[12] American College of Cardiology Foundation Appropriate Use Criteria Task Force; American Society of Echocardiography; American Heart Association; American Society of Nuclear Cardiology; Heart Failure Society of America; Heart Rhythm Society; Society for Cardiovascular Angiography and Interventions; Society of Critical Care Medicine; Society of Cardiovascular Computed Tomography; Society for Cardiovascular Magnetic Resonance, Douglas PS, Garcia MJ, Haines DE, Lai WW, Manning WJ, Patel AR, Picard MH, Polk DM, Ragosta M, Ward RP, Weiner RB (2011). ACCF/ASE/AHA/ASNC/HFSA/HRS/SCAI/SCCM/SCCT/SCMR 2011 Appropriate Use Criteria for Echocardiography. A Report of the American College of Cardiology Foundation Appropriate Use Criteria Task Force, American Society of Echocardiography, American Heart Association, American Society of Nuclear Cardiology, Heart Failure Society of America, Heart Rhythm Society, Society for Cardiovascular Angiography and Interventions, Society of Critical Care Medicine, Society of Cardiovascular Computed Tomography, and Society for Cardiovascular Magnetic Resonance Endorsed by the American College of Chest Physicians. J Am Coll Cardiol.

[13] Mohr FW, Falk V, Diegeler A, Walther T, Gummert JF, Bucerius J, Jacobs S, Autschbach R (2001). Computer-enhanced "robotic" cardiac surgery: experience in 148 patients. J Thorac Cardiovasc Surg.

[14] Murphy DA, Miller JS, Langford DA (2005). Robot-assisted endoscopic excision of left atrial myxomas. J Thorac Cardiovasc Surg.

[15] Schilling J, Engel AM, Hassan M, Smith JM (2012). Robotic excision of atrial myxoma. J Card Surg.

[16] Hassan M, Smith JM (2012). Robotic assisted excision of a left ventricular myxoma. Interact Cardiovasc Thorac Surg.

[17] Gao C, Yang M, Wang G, Wang J, Xiao C, Wu Y, Li J (2010). Excision of atrial myxoma using robotic technology. J Thorac Cardiovasc Surg.

[18] Gutiérrez de Loma J, Valderrama Marcos JF, Melero Tejedor JM, González González S (2009). Left atrial myxoma: extraction by robotic and vacuum assistance. Innovations (Phila).

[19] Watanabe G (2010). Successful intracardiac robotic surgery: initial results from Japan. Innovations (Phila).

